# What factors are associated with new social isolation years after the great East Japan Earthquake?: findings from the TMM CommCohort study

**DOI:** 10.1186/s12889-025-23778-x

**Published:** 2025-08-12

**Authors:** Yuka Kotozaki, Kozo Tanno, Kotaro Otsuka, Ryohei Sasaki, Makoto Sasaki

**Affiliations:** 1 Iwate Tohoku Medical Megabank Organization, Iwate Medical University, Yahaba, Japan; 2 Department of Hygiene and Preventive Medicine, School of Medicine, Iwate Medical University, Yahaba, Japan; 3 Department of Neuropsychiatry, School of Medicine, Iwate Medical University, Yahaba, Japan; 4 Division of Physical Education, Department of Human Sciences, Iwate Medical University Center for Liberal Arts and Sciences, Yahaba, Japan; 5 Division of Ultrahigh Field MRI, Institute for Biomedical Sciences, Iwate Medical University, Yahaba, Japan

**Keywords:** Social isolation, Related factors, Damage situation, Community-based cohort study

## Abstract

**Background:**

The effects of disaster experiences on social isolation and related factors remain unclear. Using longitudinal data, this study aimed to identify new social isolation and its associated factors after the Great East Japan Earthquake (GEJE), which occurred on March 11, 2011.

**Methods:**

We analyzed longitudinal data from 12,795 participants who responded to self-report questionnaires, utilizing the Lubben Social Network Scale-6 (LSNS-6). Participants included 4,450 men and 8,345 women; the sex ratio of the analytic sample was broadly consistent with that of the study population at baseline. Baseline data were collected between fiscal years (FY) 2013 and 2015, and follow-up data were collected between FY2017 and FY2019. Social isolation was defined as a score < 12 on the LSNS-6. Based on their level of social isolation in the second survey, participants were categorized into two groups: not socially isolated and newly socially isolated. To examine factors associated with new social isolation, we used logistic regression analysis to calculate multivariate-adjusted odds ratios (ORs) and 95% confidence intervals (CIs) for the newly socially isolated group versus the not socially isolated group by sex.

**Results:**

New social isolation was associated with several lifestyle and psychosocial factors, exhibiting differences between men and women. Among men, factors associated with new social isolation were not currently smoking among those without house damage, living alone among those with house damage, and no exercise habits among those who experienced the death of family members due to GEJE. Among women who lost family members due to GEJE, insomnia was associated with new social isolation.

**Conclusion:**

The occurrence of new social isolation years after the GEJE and its associated factors varied by sex and the presence of GEJE-related damages. To prevent new social isolation in the aftermath of a large-scale natural disaster, it is crucial to consider sex-specific factors contributing to social isolation.

**Supplementary Information:**

The online version contains supplementary material available at 10.1186/s12889-025-23778-x.

## Background

The Great East Japan Earthquake (GEJE) struck northeastern Japan on Friday, March 11, 2011, at 14:46 JST. The magnitude 9.0 earthquake was followed by a massive tsunami that caused extensive damage, particularly along the Pacific coast of the Tohoku region, such as Iwate, Miyagi, and Fukushima [1]. Approximately 15,000 people have been killed, 2,500 are missing, and 5,400 have been injured. At its peak, approximately 470,000 people were evacuated, and 406,067 buildings were reported to have been completely destroyed, washed away, or half destroyed [2]. Survivors in this area may have lost family and friends to the earthquake and tsunamis and were living in shelters after their houses were damaged; thus, they may have been at risk for social isolation.

The GEJE is considered one of the most complex natural disasters in recent history owing to its compound nature, combining an earthquake, a tsunami, and the Fukushima Daiichi nuclear accident. Unlike many other disasters, its effects were not only massive and immediate but also long lasting, with large-scale displacement, long-term reconstruction, and continuing health and environmental concerns. This prolonged disruption of everyday life makes the GEJE uniquely positioned for an examination of the long-term psychosocial consequences of disaster exposure, including social isolation [3–5].

Losing family and property to a disaster can cause severe psychological stress and trauma [6, 7]. These symptoms can make it easier to become emotionally closed off and, consequently, more difficult to connect with others and more isolated [8]. Additionally, disaster damage may force people to relocate to new areas. Owing to changes in their living environment, they lose their traditional friendships and connections with the local community. It is not easy to rebuild new relationships in a new location, and the lack of existing networks can further contribute to feelings of isolation, as there is little opportunity for emotional or physical support and no one to turn to for help [9]. Additionally, people often face economic hardship owing to unemployment or loss of livelihood following the disaster, which can significantly impact their overall lives. Financial burdens can reduce a person’s ability to afford to interact with other people and amplify feelings of isolation [10]. These various factors are considered to drive social isolation.

Social isolation refers to a lack of social contact or support [11]. Social isolation is generally acknowledged as a major public health problem [12–14]. Moreover, it is widely recognized to have detrimental health outcomes, including increased risk for poor mental health, cognitive decline, and mortality [15–17]. 

Several studies have explored the determinants of social isolation. For instance, a longitudinal study of 2,657 older residents showed that older men are more vulnerable to social isolation than women [18]. A longitudinal study of 2,641 residents aged ≥ 65 years living in a suburb of London revealed elevated risk factors for social isolation among older men, individuals living alone, and those dealing with mood or cognitive problems [19]. The Bangor Longitudinal Study of Ageing, with 20 years of follow-up, found that social isolation is associated with the death of close relatives, deteriorating health, being home alone for long periods, and lifestyle changes [20]. Previous studies indicate that sex differences influence social isolation, with women generally maintaining stronger social networks than men, who often experience greater isolation after life changes such as retirement [18, 19]. Additionally, disaster-related factors such as house damage can drastically alter living environments and social relationships [3–5, 9], especially among men, who may become more isolated following housing loss and separation from family. Furthermore, the death of family members owing to disasters has significant psychological and social consequences, increasing the risk of social isolation [6–8, 20], particularly among men who may have fewer social ties outside the home [18, 19]. Based on these findings, this study examined the interaction effects between sex, house damage, and family member death to elucidate their combined effect on new social isolation following the GEJE. Although many population-based studies have examined social isolation and its determinants, focusing primarily on the general elderly population, relatively little is known about how traumatic, large-scale events such as natural disasters influence the emergence of new social isolation. Furthermore, how these effects might differ by sex or according to the type and degree of disaster-related damage has rarely been explored.

Therefore, we hypothesized that, in addition to the traditionally identified factors associated with social isolation, specific disaster-related experiences (e.g., death of a family member, house damage) would be associated with the development of new social isolation, and that these associations would differ by sex. These factors were selected a priori based on previous literature indicating gender-based differences in social isolation vulnerability and the psychosocial impact of housing loss and bereavement after disasters. Understanding their interaction may help inform more tailored post-disaster support strategies. This study aimed to identify new social isolation and its related factors following the GEJE using the longitudinal data of the Tohoku Medical Megabank Community-based Cohort Study (TMM CommCohort Study).

## Methods

In accordance with the Declaration of Helsinki (1991), written informed consent was obtained from all participants. The Ethics Committee of Iwate Medical University (first approval: HG H25-2; most recent approval: HG 2018-004) approved all the study procedures. The study was carried out in accordance with the Declaration of Helsinki (1991).

### Participants

This study was part of the TMM CommCohort Study, which was a community-based cohort study conducted by the Iwate Tohoku Medical Megabank Organization (IMM), and a longitudinal and observational cohort study [21, 22]. The TMM CommCohort Study has been following up since 2013 to assess the long-term impact of GEJE on the affected population. Approximately 85,000 people, mostly residents of the coastal areas of Miyagi and Iwate prefectures that were severely affected by the GEJE, are participating in the study. The study collects multiple types of information, such as blood and urine samples, questionnaires, municipal health examination data (for example, height, weight, blood pressure), and physiological measurements (for example, pulse wave velocity, carotid ultrasound images). This cohort study is the only one in the world to possess such a large sample size and type of data, and to have tracked post-disaster impacts for more than 10 years. We thought that by using this cohort data, we could clarify the trajectory of new social isolation after the GEJE. This study began in July 2013, two years after the 2011 GEJE. The baseline survey data were collected between FY2013 and FY2015, and the secondary survey data were collected between FY2017 and FY2020.

Recruitment of study participants was conducted at the municipal health check-ups and our facility. Inclusion criteria were as follows: individuals aged ≥ 20 years registered in the basic resident register of all municipalities in Iwate Prefecture at the time of enrollment. A total of 32,320 out of the 32,919 participants from the baseline surveys were followed, excluding six dual enrollees and 593 individuals who withdrew their consent. Of the 32,320 participants, 24,316 participated in the secondary survey. To minimize the impact of COVID-19, of the 24,316 participants, 20,810 who participated in the secondary survey from FY2017 to FY2019 were included in the current study. Among 20,810 individuals, we excluded 210 participants who did not return the questionnaire, 1,500 participants with missing responses on the Lubben social network scale (LSNS-6) used to evaluate social isolation, as lack of this information would not allow for the assessment of social isolation status. We also excluded 1,659 participants with missing data in other variables (health status and economic factors) and were deemed to lack the necessary information for the statistical model. To focus on the incidence of new social isolation, we further excluded 4,646 participants who were socially isolated at the baseline survey. Consequently, our final analysis included 12,795 participants (4,450 men and 8,345 women; mean age at baseline survey 60.1 ± 11.1 years).

### Patient and public involvement statement

Patients and/or the public were not involved in the design, conduct, reporting, or dissemination plans of this research.

### Measures

#### Social isolation

Social isolation was assessed using the Lubben Social Network Scale-6 (LSNS-6) [13, 14]. The LSNS-6 consists of six items on social connections (3 questions about family ties and three questions about friendship ties, see Supplementary Material 2). Each item is rated on a 6-point Likert scale. The LSNS-6 ranges from 0 to 30. The reliability and validity of the Japanese version of the LSNS-6 have been confirmed [23]. A score of < 12 defines social isolation [13, 23].

### House damage, loss of family members due to the GEJE, and demographic characteristics

We used six options to assess house damage caused by the GEJE: (1) totally damaged (including all outflows); (2) seriously damaged; (3) half-damaged; (4) partially damaged; (5) no damage; and (6) non-residence. We further classified these options as damaged (totally damaged or seriously damaged, half-damaged, and partially damaged) or undamaged (no damage or non-residence). Additionally, participants responded yes or no to inquiries about the death of family members due to the GEJE.

The following demographic characteristics were included in the analysis based on strong evidence of their association with social isolation [18–20, 24]: age; sex; area (inland and coast); body mass index (BMI, < 18.5, 18.5 to < 25, or ≥ 25 kg/m^2^); education level (junior high school, high school, college or university or higher, and other); marital status (unmarried or married); the number of household members (living alone or ≥ 2); work status (unemployed or employed); income (< 2,000,000 yen, 2,000,000 to less than 4,000,000 yen, >4,000,000 yen); smoking habits (nonsmoker or current smoker); drinking habits (nondrinker or current drinker); exercise habits; depressive symptoms; insomnia; social capital; and dwelling style (prefabricated temporary/disaster restoration housing, rented housing, new homeownership after the earthquake, houses lived in before the earthquake, and other). Depressive symptoms were determined by a score of ≥ 16 on the Center for Epidemiological Studies-Depressive Scale [25, 26]. Insomnia was determined by a score of ≥ 6 on the Athens Insomnia Scale [27, 28]. Social capital was assessed by responses to the following four statements: (1) “Most people in this neighborhood are likely to help each other.” (helping each other), (2) “Most people who live in this neighborhood can be trusted.” (trust), (3) “Most people in this neighborhood are likely to greet one another.” (greeting), and (4) “If there was any problem in this community, how likely is it that people will cooperate to try to solve the problem?” (solving problems together). The subjects were asked to choose one of the following responses: “strongly agree,” “somewhat agree,” “neither agree nor disagree,” “somewhat disagree,” and “strongly disagree.” Responses were scored, and a total score was calculated for the four questions [29]. Lack of social capital was determined by a score of < 9, based on previous studies [30, 31]. Social network is a structural concept that represents who and how many people an individual is connected to, whereas social capital is related to the quality and value of relationships, focusing on the trust and support generated through these connections. Since these two variables capture different aspects, we included both in this study. House damage, death of family members due to the GEJE, and educational level were asked only in the baseline survey. Income was asked only in the second survey. Other variables were asked in both baseline and second surveys, and the answered values from the second survey were used.

### Statistical analysis

Based on their level of social isolation in the second survey (LSNS-6 < 12), participants were categorized into two groups: not socially isolated (LSNS-6 ≥ 12) and newly socially isolated (LSNS-6 < 12). We used t-tests for continuous variables and the chi-square test for categorical variables to compare the characteristics of the two groups. We also performed a Fisher’s z-transformation to examine the between-group within-sex correlations of the LSNS-6 scores.

To examine factors associated with new social isolation, we used logistic regression analysis to calculate multivariate-adjusted odds ratios (ORs) and 95% confidence intervals (CIs) for the newly socially isolated group versus the not socially isolated group by sex. The not socially isolated group was treated as the reference. The following variables were entered in the multivariate adjusted model: age, area, BMI, education level, marital status, the number of household members, work status; income, smoking habits, drinking habits, exercise habits, depressive symptoms, insomnia, social capital, dwelling style, house damage, and loss of family members due to the GEJE. Stratified analysis was also conducted to examine the effects of age and damage owing to the effect of GEJE (house damage or the death of family members).

Furthermore, to determine whether the effects on the association between social isolation and each factors differed depending on the presence or absence of experiences such as house damage or the death of family members due to the GEJE, specifically interaction terms were created between house damage or the death of family members due to the GEJE and each factor (for example, house damage*smoking habits). We used a logistic regression model that included both main effects and interaction terms (for example, house damage, smoking habits, drinking habits, house damage*smoking habits, and house damage*drinking habits). The interaction term provides a more detailed understanding of the increased risk of social isolation when certain factors are combined, a relationship not captured by the main effect alone.

Finally, as part of a sensitivity analysis, we evaluated the impact of missing data by performing multiple imputation using the fully conditional specification method. The results exhibited no significant differences between the imputed dataset and the complete-case analysis. Therefore, we selected the complete-case analysis as the primary approach, as it enables statistical analysis to be conducted more quickly and efficiently.

All statistical analyses were conducted using SPSS version 25.0 for Windows (IBM, Tokyo, Japan). *P* values of < 0.05 indicated statistical significance.

## Results

The LSNS-6 reliability for this sample was 0.83 for Cronbach’s alpha coefficient and 0.71 (95% CI 0.68 to 0.73) for the within-class phase coefficient. Factor structure data for LSNS-6 are presented in Supplemental Table 1. The three family items all load heavily on that factor. Additionally, the three friend/neighbor items also load heavily on the friend/neighbor factor. The eigenvalues suggest a strong principal component. Supplemental Table 2 shows the between-group within-sex correlations of the LSNS-6 scores by sex.

The baseline and secondary survey characteristics of participants across the two social isolation groups are presented in Tables 1 and 2. Compared with the not socially isolated group, the newly socially isolated group demonstrated several notable differences. At both time points, individuals in the newly isolated group tended to be younger, had higher rates of being unmarried, and were more likely to report no exercise habits, depressive symptoms, and insomnia. For example, at baseline, the proportion of unmarried individuals was higher in the newly isolated group (men: 17.7% vs. 13.0%; women: 24.8% vs. 21.5%), and the prevalence of depressive symptoms was also elevated (men: 20.5% vs. 14.4%; women: 33.8% vs. 22.3%). These patterns remained consistent in the secondary survey (e.g., insomnia in women: 32.8% vs. 24.0%). By contrast, employment and drinking status were relatively comparable between the newly and not socially isolated groups (e.g., baseline unemployment in men: 37.4% vs. 36.5%; current drinking in women: 37.2% vs. 34.6%). However, the prevalence of current smoking showed a divergent pattern: Women in the newly isolated group had a higher prevalence of current smoking, whereas men had a slightly lower prevalence, although these differences were not statistically significant. Overall, these findings suggest that psychosocial and health-related characteristics—particularly marital status, mental health, and health behaviors such as physical activity—may play a more important role in the onset of new social isolation following a disaster than employment or alcohol habits. However, it should be noted that the relationship between mental health and social isolation may be bidirectional; social isolation may both result from and exacerbate poor mental health, including depressive symptoms.

**Table 1:** Comparison of baseline characteristics between not socially isolated and newly socially isolated groups

		Men			Women		
		Not socially isolated	Newly socially isolated	*p* value	Not socially isolated	Newly socially isolated	*p* value
n		3,637	813		7,041	1,304	
Age in baseline surveys (SD)		62.4 (10.8)	60.7 (10.9)	< 0.001	59.4 (11.0)	56.7 (11.4)	< 0.001
Area (%)	Coast	79.5	74.9	0.004	81.4	78.2	0.022
BMI (%)	< 18.5	1.3	2.8	0.008	6.4	9.2	< 0.001
	≥ 18.5–<25.0	62.4	62.6		67.7	66.6	
	≥ 25.0	36.3	34.6		25.9	24.2	
Education level (%)	Junior high school	23.3	19.1	0.051	18.4	16.3	0.066
	High school	48.8	50.3		47.2	49.6	
	College/university or higher	26.9	29.8		33.4	33.8	
	Other	1.0	0.9		1.0	0.3	
Marital status (%)	Unmarried	13.0	17.7	< 0.001	21.5	24.8	0.005
Number of household members (%)	Alone	5.4	7.5	0.018	8.6	8.6	0.952
Work status (%)	Unemployed	36.5	37.4	0.638	48.7	48.0	0.525
Smoking habits (%)	Current smoker	25.4	22.4	0.074	4.7	7.9	< 0.001
Drinking habits (%)	Current drinker	77.2	87.6	0.373	34.6	37.2	0.149
Exercise habits (%)	No	52.7	57.4	0.014	56.1	63.0	< 0.001
Depressive symptoms (%)		14.4	20.5	< 0.001	22.3	33.8	< 0.001
Insomnia (%)		14.5	18.2	0.009	21.7	28.0	< 0.001
Social capital (%)		0.6	2.0	< 0.001	0.4	1.2	< 0.001
Dwelling style (%)	Prefabricated temporary housing/Disaster restoration housing	6.2	6.2	0.774	6.7	6.8	0.019
	Rented housing	4.4	5.3		5.2	6.6	
	New homeownership after the earthquake	7.9	8.0		8.0	7.4	
	Houses lived in before the earthquake	78.7	77.4		77.1	76.0	
	Other	2.7	3.2		3.0	3.1	
House damage due to the GEJE		34.6	32.8	0.335	34.4	31.9	0.081
The death of family members due to the GEJE		3.3	3.2	0.853	4.4	4.6	0.906

*SD* Standard deviation, *BMI* Body mass index. t-test for group comparisons of continuous variables, and chi-square test for categorical variable were performed

Statistical significance: *P* < 0.05

**Table 2:** Comparison of secondary survey’s characteristics between not socially isolated and newly socially isolated groups

		Men			Women		
		Not socially isolated	Newly socially isolated	*p* value	Not socially isolated	Newly socially isolated	*p* value
n		3,637	813		7,041	1,304	
Age		66.6 (10.8)	64.9 (10.9)	< 0.001	63.5 (11.0)	60.9 (11.4)	< 0.001
BMI (%)	≥ 18.5	1.4	2.7	0.036	6.1	7.4	< 0.001
	≥ 18.5, < 25.0	59.3	58.3		64.5	65.9	
	≥ 25.0	39.2	39.0		29.4	26.7	
Marital status (%)	Unmarried	13.5	18.7	< 0.001	24.7	26.6	0.077
Number of household members (%)	Alone	6.2	9.3	0.001	11.5	11.3	0.900
Work status (%)	Unemployed	39.9	39.2	0.729	50.8	45.2	< 0.001
Income (%)	Less than 2,000,000	17.8	21.3	0.055	23.3	25.8	0.001
	2,000,000 to less than 4,000,000	46.1	45.5		40.0	43.3	
	More than 4,000,000	36.0	33.2		36.8	30.9	
Smoking habits (%)	Current smoker	25.4	22.4	0.077	4.5	7.8	< 0.001
Drinking habits (%)	Current drinker	74.0	75.0	0.537	32.4	35.4	0.089
Exercise habits (%)	No	51.4	59.7	< 0.001	54.8	64.9	< 0.001
Depressive symptoms (%)		15.3	26.2	< 0.001	22.5	38.1	< 0.001
Insomnia (%)		15.5	25.4	< 0.001	24.0	32.8	< 0.001
Social capital (%)		0.6	1.7	< 0.001	0.5	2.4	< 0.001
Dwelling style (%)	Prefabricated temporary housing/Disaster restoration housing	2.4	2.0	0.092	3.1	3.5	< 0.001
	Rented housing	4.7	6.8		6.0	8.4	
	New homeownership after the earthquake	14.4	12.8		14.1	13.1	
	Houses lived in before the earthquake	73.9	73.2		71.8	70.0	
	Other	4.6	5.2		4.9	5.0	

*BMI* Body mass index. t-test for group comparisons of continuous variables and chi-square test for categorical variable were performed

Statistical significance: *P* < 0.05

Corresponding results based on the multiple imputation dataset are presented in Supplementary Tables S3 and S4. The distributions across groups were comparable to those observed in the complete-case dataset, supporting the robustness of the main findings.

Table 3 displays the multivariable-adjusted ORs for the newly socially isolated group compared to the not socially isolated group. For both men and women, the tendency in factors associated with newly social isolation was generally the same. Non-significant variables were excluded from the main tables for clarity but are shown in Supplementary Table 5. A significant interaction effect by sex on social isolation was confirmed in current smoking (OR [95%CI] = 0.75 [0.62-0.91] for men, OR = 1.47 [1.15-1.87] for women, p for interaction < 0.001), and insomnia (OR = 1.49 [1.22-1.83] for men, OR = 1.15 [0.99-1.33] for women, p for interaction = 0.026). Sensitivity analyses using the multiply imputed dataset yielded comparable results, reinforcing the robustness of the findings. These results are presented in Supplementary Table 6.

**Table 3:** Multivariable-adjusted odds ratio of newly socially isolated group to not socially isolated group

		Reference	Men (*n* = 813)	Women (*n* = 1,304)	*P* for interaction
Smoking habits	Current smoker	none	0.75 (0.62-0.91)	1.47 (1.15-1.87)	*p* < 0.001
Insomnia	≥ 6	< 6	1.49 (1.22-1.83)	1.15 (0.99-1.33)	*p* = 0.026

Statistical significance: *P* < 0.05

Only significant interaction terms (*p* < 0.05) are presented. Non-significant variables were excluded from the main tables for clarity but are shown in Supplementary Table 5

The multivariable-adjusted ORs of the newly socially isolated group to the not socially isolated group by age group are in Supplemental Table 7. Among men, a significant interaction effect by age group on social isolation was confirmed in those with low income (OR = 1.93 [1.23-3.03] for men under 65 years old, 1.40 [1.09-1.81] for men over 65 years old, p for interaction = 0.025). Among women, a significant interaction effect by age group on social isolation was confirmed in those who were current smoking (OR = 1.20 [0.90-1.58] for women under 65 years old, OR = 2.81 [1.72-4.59] for women over 65 years old, p for interaction = 0.003).

Table 4 presents the multivariable-adjusted ORs for the newly socially isolated group compared to the not socially isolated group by house damage. Non-significant variables were excluded from the main tables for clarity but are shown in Supplementary Table 8. Among men, a significant interaction effect by house damage on social isolation was confirmed in those who were living alone (damage: OR = 1.17 [0.94-1.49] vs. no damage: OR = 1.96 [1.04-3.66], p for interaction = 0.020) and current smoking (damage: OR = 0.65 [0.51-0.83] vs. no damage: OR = 0.96 [0.70-1.31], p for interaction = 0.042).

**Table 4:** Multivariable-adjusted odds ratio of newly socially isolated group to not socially isolated group by house damage

			Men (*n* = 813)			Women (*n* = 1,304)		
		Reference	Undamaged (*n* = 546)	Damaged (*n* = 267)	P for interaction	Undamaged (*n* = 888)	Damaged (*n* = 416)	P for interaction
Number of household members	Alone	Two or more	1.17 (0.77-1.79)	1.96 (1.04-3.66)	*p* = 0.020	0.90 (0.67-1.20)	0.82 (0.54-1.24)	*p* = 0.764
Smoking habits	Current smoker	none	0.65 (0.51-0.83)	0.96 (0.70-1.31)	*p* = 0.042	1.67 (1.24-2.26)	1.14 (1.04-1.70)	*p* = 0.115

Statistical significance: *P* < 0.05

Note: Only significant interaction terms (*p* < 0.05) are presented. Non-significant variables were excluded from the main tables for clarity but are shown in Supplementary Table 8

Finally, Table 5 displays the multivariable-adjusted ORs for the newly socially isolated group compared to the not socially isolated group by the death of family members due to GEJE. Non-significant variables were excluded from the main tables for clarity but are shown in Supplementary Table 9. Among men, a significant interaction effect by the death of family members due to GEJE on social isolation was confirmed in those without exercise habits (No family death: OR = 1.26 [1.06-1.50] vs. Family death OR = 6.51 [1.66-25.52] for men who have experienced the death of a family member, p for interaction = 0.021). Among women, a significant interaction effect by the death of family members due to GEJE on social isolation was confirmed in those with insomnia (No family death: OR = 1.11 [0.95-1.28] vs. Family death: OR = 2.63 [1.32-5.24], p for interaction = 0.009). Some ORs in Table 5, such as for men who lost a family member and lacked exercise (OR = 6.51, 95% CI: 1.66–25.52), had wide confidence intervals, indicating limited precision. These findings should be interpreted with caution.

**Table 5:** Multivariable-adjusted odds ratio of newly socially isolated group to not socially isolated group by the death of family members due to the GEJE

			Men (*n* = 813)			Women (*n* = 1,304)		
		Reference	No experience (*n* = 787)	Experience (*n* = 26)	P for interaction	No experience (*n* = 1,244)	Experience (*n* = 60)	P for interaction
Exercise habits	No	Yes	1.26 (1.06-1.50)	6.51 (1.66-25.52)	*p* = 0.021	1.36 (1.18-1.56)	1.60 (0.81-3.16)	*p* = 0.492
Insomnia	≥ 6	< 6	1.49 (1.21-1.83)	1.36 (0.31-5.99)	*p* = 0.693	1.11 (0.95-1.28)	2.63 (1.32-5.24)	*p* = 0.009

*GEJE* Great east Japan earthquake

Statistical significance: *P* < 0.05

Only significant interaction terms (*p* < 0.05) are presented. Non-significant variables were excluded from the main tables for clarity but are shown in Supplementary Table 9. ORs that are extremely high or low should be interpreted with caution, as they may reflect small sample sizes or the influence of outliers

## Discussion

We demonstrated the factors associated with new social isolation among the residents affected by GEJE. While many of these factors were common to both men and women, there were some notable differences. For instance, smoking habits were significantly associated with a decreased risk of new social isolation in men but an increased risk in women. Living alone was associated with new social isolation in men who had experienced house damage, whereas not smoking was related to new social isolation in men who had not experienced house damage. Not having exercise habits was linked to a higher risk of new social isolation, particularly among men who had experienced the death of family members due to the GEJE. Insomnia was associated with new social isolation for women who had experienced the death of a family member due to the GEJE.

The LSNS-6 reliability for this sample was 0.83 for Cronbach’s alpha coefficient and 0.71 (95% CI 0.68 to 0.73) for the within-class phase coefficient. This result is almost identical to the Cronbach’s alpha coefficient in the results of Kurimoto et al. Although the intraclass correlation coefficient is lower than that of Kurimoto et al. (0.96), our results are considered similar in trend, as the correlation coefficients are interpreted as weak (0.1–0.3), moderate (0.4–0.6) and strong (> 0.7) [32]. Additionally, the LSNS-6 factor structure for this sample had an eigenvalue of 3.5 for the family factor (both baseline and second survey) and 1.2 for the friends/neighbor factor (both baseline and second survey). These results are similar to those of Lubben et al. (2006), which suggests that the LSNS-6 is being measured appropriately [14].

Generally, women tend to have stronger links with friends and family, and more developed support networks [33]. Particularly, among older people, women interact frequently with friends and family and often seek support to alleviate feelings of social isolation [34]. In contrast, men tend to be at greater risk of social isolation because their work is often the center of their lives and their social connections suddenly decline after retirement, often limiting their support networks [33]. 

In our study, we found a significant association between current smoking and new social isolation among women, especially those aged 65 or older. It has been reported that women’s social isolation is significantly associated with smoking habits and is more pronounced among older women [35, 36], and our results support previous findings. In some cultures, it is considered inappropriate for women to smoke. This social stigma can contribute to the isolation of women smokers from their environment [37]. Particularly in areas with strong traditional values, women who smoke are at high risk of being ostracized by their families and communities [37]. Female smokers are also more likely to lose contact with others, with limited opportunities for support owing to social stigma. This isolation can create a vicious cycle that increases smoking [38]. In addition, in later life, the physical consequences of smoking, particularly pulmonary impairment, may reduce physical capacity and further limit opportunities for social engagement [39]. 

In contrast to women, for whom smoking may carry social stigma and thus contribute to isolation, smoking among men—especially in the Japanese sociocultural context—may serve a fundamentally different function. We observed an inverse association between current smoking and the occurrence of social isolation among men, particularly among those who had not experienced house damage due to the GEJE. Previous studies have demonstrated that social isolation is associated with health-risk behaviors such as smoking [40, 41]. In some societies, smoking may be considered a behavior that symbolizes ‘masculinity’ and assertiveness [42, 43]. This expectation may contribute to higher rates of smoking among men; simultaneously, smoking may be chosen as a means of avoiding social isolation. Social smoking tendency is more pronounced in older generations in Japan. Smoking has long been embedded in male workplace culture and interpersonal relationships, where smoking areas have traditionally functioned as spaces for social interaction [36, 44, 45]. Such sociocultural factors may reinforce the association between smoking and the maintenance of social connections among men. These contrasting associations between smoking and social isolation in men and women may reflect gender-specific sociocultural norms and stigma. For men, smoking may function as a facilitator of social interaction, particularly in the workplace or in male peer groups, where it is perceived as normative or even masculine. By contrast, for women, smoking may serve as a marker of social deviance, attracting stigma and reducing opportunities for supportive interaction. Thus, the same behavior—smoking—can have opposite social consequences depending on gender, potentially explaining the divergent associations observed in this study.

DeWall et al. proposed that smoking can provide a sense of social belonging [46]. This sense of belonging may stem from the role smoking plays in structuring social interactions, maintaining identity with smoking companions, and fostering conversation during smoking [47, 48]. While most smokers smoke to relieve stress or alter their mood, some smokers use it as a means of engaging in social discourse during these moments [49]. Against this backdrop, smoking among men may be viewed as a form of communication or social bonding, particularly within the Japanese cultural context.

Insomnia was associated with new social isolation among men in our study. Insomnia is a well-documented concern among individuals experiencing social isolation [50, 51]. McLay et al. identified an association between social isolation and insomnia in men using data from a national cohort in New Zealand [51]. Men are often culturally expected to refrain from emotional expression and may be less likely to share insomnia and its effects with others [52]. They may feel more isolated because persistent insomnia can reduce their work productivity and lead to a loss of social roles. In addition, we indicated that new social isolation was associated with insomnia among women who experienced the loss of a family member due to the GEJE. Monk et al. similarly reported that family bereavement could trigger insomnia in women [53]. What is particularly noteworthy is that the effects of family bereavement on women’s insomnia may extend beyond the immediate aftermath, persisting for years [53]. Losing a family member is very stressful. The experience of suddenly losing a family member following a disaster is unimaginably stressful, and the grief is usually longer than expected. While it is often an acute grief reaction or a grief that lasts from one year to several years, some cases of grief can persist for years in the form of complicated grief [54]. A study that examined the trajectory of grief over a six-year period following a natural disaster reported that 48% of the total sample was recovering from grief, and 11% was chronically grieving [55]. Women tend to be more stressed than men [56]. Chronic stress can increase the risk of developing insomnia [57]. Persistent insomnia can lead to isolation, as lack of energy and concentration can make it difficult to maintain social relationships [58]. It is imperative to be vigilant regarding insomnia, especially among women who have lost family members in the wake of a disaster, as it can potentially lead to social isolation.

Living alone was associated with new social isolation among men who had experienced house damage due to the GEJE. However, this association was not observed in women. Men typically have smaller social networks and less regular contact with children and relatives over the course of their lives. Furthermore, men are at increased risk of isolation in the event of divorced or becoming widowed [59]. Indeed, living alone and not being in a relationship with a partner are substantial risk factors for social isolation [60, 61]. Specific social environments also foster social isolation [62]. Men who experienced house damage as a result of the earthquake often transitioned to living alone owing to the rapid changes in their environment caused by the loss of their housing and separation from their families [63], which may have driven new social isolation.

Not having exercise habits was found to be associated with a higher risk of new social isolation, particularly among men who had experienced the loss of family members due to the GEJE. An association between lack of exercise habits and social isolation has been reported [64, 65]. After death of family members, remaining family members most directly affected will suffer detrimental effects on their physical or mental health, or both [66]. Considering that men often have few social ties outside work and home and are more likely to become socially isolated when their relationships with family and neighbors fade, losing a family member may lead to social isolation owing to apathy and reduced contact with the community and other people as they refrain from outdoor activities.

Among men under the age of 65, our study revealed a notably elevated risk of new social isolation associated with low income. Men’s social isolation is associated with low income [67, 68]. An extensive analysis of a substantial dataset from the UK Biobank, comprising approximately 400,000 adults, underscores that men with lower incomes are more likely to report experiencing social isolation compared to women. Men’s income strongly predicts the frequency with which they interact with (or visit) friends and family [68]. Low-income men find it difficult to find money for hobbies and entertainment. This financial constraint can compel low-income men to curtail their social engagements, thereby exacerbating their sense of isolation.

Despite the well-established observation that men tend to experience more social isolation than women [69, 70], sex-specific patterns of social isolation have received relatively limited attention and have only recently begun to be explored in greater detail [70]. Our findings contribute significantly to the growing body of knowledge regarding gender-based patterns of social isolation, potentially offering valuable insights for identifying and supporting individuals who are most vulnerable to social isolation. These gender-specific associations may be understood within the broader sociocultural context of Japan. In Japanese society, gender roles have traditionally been well defined, with men often expected to assume the role of breadwinners and women as caretakers within the household [71]. These expectations shape the ways in which men and women engage with others, seek support, and cope with adversity. For instance, men are more likely to derive their identity and social connections from their workplace, and they may lack alternative social networks once they retire or leave the workforce [72]. By contrast, women tend to form and maintain diverse social relationships through family, neighborhood, or community activities [73]. After a disaster such as the GEJE, these sociocultural patterns may amplify gender differences in vulnerability to social isolation [74]. Men who lose their homes or jobs may become socially disengaged owing to the loss of role and purpose, while women may rely on their interpersonal ties to seek emotional support. Understanding these culturally embedded gender norms is essential for interpreting the observed sex differences in the associations between disaster-related experiences, health behaviors, and social isolation. These sociocultural patterns also highlight the importance of designing post-disaster interventions that are responsive to specific gender-based vulnerabilities, such as providing sleep-focused psychological support for bereaved women or social engagement programs for isolated men living alone.

Furthermore, there is a pressing need for long-term research aimed at providing effective support to prevent social isolation, regardless of whether individuals have been affected by past earthquakes or other adverse events. Our study’s results complement prior research on social isolation and its associated factors, which has predominantly been conducted in Europe and the United States. This underscores the importance of future research endeavors focused on investigating social isolation, psychosocial determinants, and health-related behaviors in diverse contexts.

The present study has some limitations. First, this study excluded 1,659 participants because of missing data on covariates. Although this approach is commonly used, it may have introduced selection bias, especially if the missing data were not completely random. For example, if those with missing data differed systematically in exposure or outcome status, the results may be biased. Hence, these findings should be interpreted cautiously. Additionally, this study is based on the results of a survey of residents who participated in the Tohoku Medical Megabank CommCohort Study in Iwate Prefecture, and there are some residents who could not participate in the health survey. Consequently, the results may have underestimated the prevalence of social isolation, as those who participated in the health survey might have exhibited a higher degree of health awareness and possessed the physical and mental strength necessary to attend the survey venue. This could have introduced survivor bias, whereby healthier or more socially connected individuals were more likely to be included, while those with poor health or greater social isolation may have been excluded. Second, all information collected in this study, including data on the extent of damage immediately following the earthquake, was self-reported; therefore, the results may have been underestimated or overestimated. This introduces the possibility of recall bias, particularly regarding retrospective reports of disaster-related experiences and psychosocial factors. Third, some ORs, particularly those involving interaction effects, showed wide confidence intervals, indicating the limited precision of the estimates. For example, the OR for men who lost a family member due to the GEJE and lacked exercise was 6.51 (95% CI: 1.66–25.52), suggesting a potential association but with substantial statistical uncertainty. This may reflect small sample sizes in certain subgroups or the influence of outliers. Therefore, these findings should be interpreted with caution. Fourth, we cannot rule out that these findings may be a consequence of conducting multiple comparisons. However, it is worth highlighting the significance of this study, as there are few known investigations that have explored the relationship between changes in social isolation and related factors following an earthquake using a population-based cohort study design with a large sample size. Fifth, although we adjusted for several relevant variables, we cannot rule out the possibility of residual confounding. For example, unmeasured factors such as baseline health status, psychological resilience, or changes in social support networks over time may have influenced both disaster-related experiences and the risk of developing social isolation. Finally, information regarding people who may have moved to this area from other areas after the GEJE was unavailable because this study was conducted two years after the GEJE.

In conclusion, our study of social isolation and its associated factors following the GEJE revealed that certain factors were common for both men and women. Nevertheless, there were variations in the relevant factors identified for men and women. Therefore, to prevent new social isolation after a large-scale natural disaster, it is necessary to provide continuous psychosocial support to disaster victims, both in terms of community-level interventions and government policies, thereby discouraging them from becoming socially isolated from their local communities. Community-based activities should be designed with careful consideration of gender differences and disaster exposure levels, as these factors were shown to influence the risk of social isolation in this study. For example, women who have lost family members and are experiencing insomnia may benefit from targeted psychological support, such as cognitive behavioral therapy for insomnia, grief counseling, and peer-based support groups tailored to bereaved women. These interventions should be accessible through local health centers or community-based outreach to ensure they reach vulnerable individuals. For men who live alone and experienced house damage, social reintegration programs could be particularly effective—such as organized volunteer activities, community leadership roles, or “men’s groups” that provide opportunities for interaction and shared purpose. Among men under 65 with low income, financial barriers may exacerbate social isolation. Therefore, policies should include free or low-cost access to community centers, digital inclusion initiatives, and subsidized participation in group-based hobbies or cultural activities that foster social connection. Finally, for men who have lost family members and lack exercise habits, local governments and municipalities could implement structured group exercise programs, such as walking groups or community sports events, which can improve both mental well-being and social connectivity. Regular community events should ensure inclusivity across gender and age, with attention to physical accessibility, mental health status, and family situation to reach those at greatest risk of isolation. In addition to the provision of temporary housing and reconstruction housing, government support should also include relocation assistance, mental health services, job retraining programs, and digital literacy training to help disaster victims adapt to changing social environments and maintain social connections. Importantly, policymakers should recognize that factors contributing to social isolation differ by sex and disaster impact and tailor interventions accordingly to achieve more effective and equitable support. These multifaceted efforts can not only prevent social isolation but also facilitate more inclusive and resilient recovery from disasters.

## Supplementary Information

Supplementary Material 1.

Supplementary Material 2.

## Data Availability

The TMM cohort data are available for a fee to researchers in Japan who have been approved by the prescribed registration and review procedures. For more information, visit: http://www.dist.megabank.tohoku.ac.jp/.

## Abbreviations

GEJE: Great East Japan Earthquake
LSNS-6: Lubben Social Network Scale-6
ORs: Odds ratios
CIs: Confidence intervals
TMM CommCohort Study: Tohoku Medical Megabank Community-based Cohort Study
IMM: Iwate Tohoku Medical Megabank Organization
BMI: Body mass index
