# Supplementary material for: What factors are associated with new social isolation years after the great East Japan Earthquake?: findings from the TMM CommCohort study
Source: BMC Public Health. 2025 Aug 12;25:2745. doi: 10.1186/s12889-025-23778-x (PMC12341258; doi:10.1186/s12889-025-23778-x)
Supplement: Supplementary file 1 — Supplementary Material 1. [file 12889_2025_23778_MOESM1_ESM.docx]

**Supplemental Table 1. The LSNS-6 Factor Matrix**

|  | Baseline survey | | Second survey | |
| --- | --- | --- | --- | --- |
|  | Family factor | Friend factor | Family factor | Friend factor |
| LSNS-6 Items |  |  |  |  |
| SNQ1 Family: size | 0.80 | 0.14 | 0.81 | 0.16 |
| SNQ2 Family: discuss private matters | 0.87 | 0.26 | 0.88 | 0.26 |
| SNQ3 Family: call for help | 0.85 | 0.26 | 0.86 | 0.25 |
| SNQ4 Friend/neighbor: size | 0.18 | 0.82 | 0.19 | 0.84 |
| SNQ5 Friend/neighbor: discuss private matters | 0.23 | 0.90 | 0.24 | 0.90 |
| SNQ6 Friend/neighbor: call for help | 0.25 | 0.88 | 0.26 | 0.88 |
| Eigenvalues | 3.5 | 1.2 | 3.5 | 1.2 |
| Percent of variability explained | 57.9% | 20.2% | 59.4% | 20.0% |

**Supplemental Table 2. The between-group within-sex correlations of the LSNS-6 scores by sex**

|  | Group1 | Group2 | r1 | r2 | z1 | z2 | z-value | p-value |
| --- | --- | --- | --- | --- | --- | --- | --- | --- |
| Not socially isolated | Men | Women | 0.53 | 0.56 | 0.59 | 0.63 | -2.09 | 0.04 |
| Newly socially isolated | Men | Women | 0.06 | 0.11 | 0.06 | 0.11 | -1.13 | 0.26 |

**Supplemental Table 3. Participants’ baseline characteristics according to the four groups of social isolation after multiple imputation**

|  | | Men | | | Women | | |
| --- | --- | --- | --- | --- | --- | --- | --- |
|  | | Not socially isolated | Newly socially isolated | *p* value | Not socially isolated | Newly socially isolated | *p* value |
| n | | 3,948 | 883 |  | 7,629 | 1,421 |  |
| Age in baseline surveys (SD) | | 62.4 (10.8) | 60.7 (10.9) | <0.001 | 59.4 (11.0) | 56.7 (11.4) | <0.001 |
| Area (%) | Coast | 79.5 | 74.9 | 0.013 | 81.4 | 78.2 | 0.035 |
| BMI (%) | <18.5 | 1.3 | 2.8 | 0.035 | 6.4 | 9.2 | <0.001 |
|  | ≥18.5–<25.0 | 62.4 | 62.6 |  | 67.7 | 11.5 |  |
|  | ≥25.0 | 36.3 | 34.6 |  | 25.9 | 24.2 |  |
| Education level (%) | Junior high school | 23.3 | 19.1 | 0.001 | 18.4 | 16.3 | 0.024 |
|  | High school | 48.8 | 50.3 |  | 47.2 | 49.6 |  |
|  | College/university or higher | 26.9 | 29.8 |  | 33.4 | 33.8 |  |
|  | Other | 1.0 | 0.9 |  | 1.0 | 0.3 |  |
| Marital status (%) | Unmarried | 13.0 | 17.7 | <0.001 | 21.5 | 24.8 | <0.001 |
| Number of household members (%) | Alone | 5.4 | 7.5 | <0.001 | 8.6 | 8.6 | 0.991 |
| Work status (%) | Unemployed | 36.5 | 37.4 | 0.041 | 48.7 | 48.0 | 0.019 |
| Smoking habits (%) | Current smoker | 25.4 | 22.4 | 0.147 | 4.7 | 7.9 | <0.001 |
| Drinking habits (%) | Current drinker | 77.2 | 87.6 | <0.001 | 34.6 | 37.2 | 0.05 |
| Exercise habits (%) | No | 52.7 | 57.4 | <0.001 | 56.1 | 63.0 | <0.001 |
| Depressive symptoms (%) |  | 14.4 | 20.5 | <0.001 | 22.3 | 33.8 | <0.001 |
| Insomnia (%) |  | 14.5 | 18.2 | <0.001 | 21.7 | 28.0 | <0.001 |
| Social capital (%) |  | 0.6 | 2.0 | <0.001 | 0.4 | 1.2 | <0.001 |
| Dwelling style (%) | Prefabricated temporary housing/ Disaster restoration housing | 6.2 | 6.2 | 0.021 | 6.7 | 6.8 | 0.01 |
|  | Rented housing | 4.4 | 5.3 |  | 5.2 | 6.6 |  |
|  | New homeownership after the earthquake | 7.9 | 8.0 |  | 8.0 | 7.4 |  |
|  | Houses lived in before the earthquake | 78.7 | 77.4 |  | 77.1 | 76.0 |  |
|  | Other | 2.7 | 3.2 |  | 3.0 | 3.1 |  |
| House damage due to the GEJE |  | 34.6 | 32.8 | 0.892 | 34.4 | 31.9 | 0.241 |
| The death of family members due to the GEJE |  | 3.3 | 3.2 | 0.129 | 4.4 | 4.6 | 0.501 |

SD: standard deviation; BMI: body mass index.
t-test for group comparisons of continuous variables, and the chi-square test was used for categorical variables.
Statistical significance was set at P < 0.05.
*n* values may slightly differ due to imputation.

**Supplemental Table 4. Characteristics of the participants according to the four groups of social isolation in the second survey after multiple imputation**

|  | | Men | | | Women | | |
| --- | --- | --- | --- | --- | --- | --- | --- |
|  | | Not socially isolated | Newly socially isolated | *p* value | Not socially isolated | Newly socially isolated | *p* value |
| n | | 3,948 | 883 |  | 7,629 | 1,421 |  |
| Age | | 66.6 (10.8) | 64.9 (10.9) | <0.001 | 63.5 (11.0) | 60.9 (11.4) | <0.001 |
| BMI (%) | ≥18.5 | 1.4 | 2.7 | 0.039 | 6.1 | 7.4 | 0.01 |
|  | ≥18.5, <25.0 | 59.3 | 58.3 |  | 64.5 | 65.9 |  |
|  | ≥25.0 | 39.2 | 39.0 |  | 29.4 | 26.7 |  |
| Marital status (%) | Unmarried | 13.5 | 18.7 | <0.001 | 24.7 | 26.6 | 0.001 |
| Number of household members (%) | Alone | 6.2 | 9.3 | <0.001 | 11.5 | 11.3 | 0.674 |
| Work status (%) | Unemployed | 39.9 | 39.2 | 0.015 | 50.8 | 45.2 | <0.001 |
| Income (%) | Less than 2,000,000 | 17.8 | 21.3 | <0.001 | 23.3 | 25.8 | <0.001 |
|  | 2,000,000 to less than 4,000,000 | 46.1 | 45.5 |  | 40.0 | 43.3 |  |
|  | More than 4,000,000 | 36.0 | 33.2 |  | 36.8 | 30.9 |  |
| Smoking habits (%) | Current smoker | 25.4 | 22.4 | 0.143 | 4.5 | 7.8 | <0.001 |
| Drinking habits (%) | Current drinker | 74.0 | 75.0 | <0.001 | 32.4 | 35.4 | 0.006 |
| Exercise habits (%) | No | 51.4 | 59.7 | <0.001 | 54.8 | 64.9 | <0.001 |
| Depressive symptoms (%) |  | 15.3 | 26.2 | <0.001 | 22.5 | 38.1 | <0.001 |
| Insomnia (%) |  | 15.5 | 25.4 | <0.001 | 24.0 | 32.8 | <0.001 |
| Social capital (%) |  | 0.6 | 1.7 | <0.001 | 0.5 | 2.4 | <0.001 |
| Dwelling style (%) | Prefabricated temporary housing/ Disaster restoration housing | 2.4 | 2.0 | <0.001 | 3.1 | 3.5 | 0.013 |
|  | Rented housing | 4.7 | 6.8 |  | 6.0 | 8.4 |  |
|  | New homeownership after the earthquake | 14.4 | 12.8 |  | 14.1 | 13.1 |  |
|  | Houses lived in before the earthquake | 73.9 | 73.2 |  | 71.8 | 70.0 |  |
|  | Other | 4.6 | 5.2 |  | 4.9 | 5.0 |  |

SD: standard deviation; BMI: body mass index.
t-test for group comparisons of continuous variables, and the chi-square test was used for categorical variables.
Statistical significance was set at P < 0.05.
*n* values may slightly differ due to imputation.

**Supplemental Table 5. Results for variables excluded from Table 3 due to lack of statistical significance (p ≥ 0.05)**

|  | | Reference | Men (n = 813) | Women (n = 1,304) | P for  interaction |
| --- | --- | --- | --- | --- | --- |
| Age group | 65 or more | Less than 65 | 0.61 (0.48－0.78) | 0.73 (0.62－0.84) | p=0.812 |
| Area | Coast | Inland | 0.82 (0.64－1.06) | 0.79 (0.67－1.00) | p=0.388 |
| BMI | <18.5 | ≥18.5, <25.0 | 2.07 (1.23－3.50) | 1.10 (0.87－1.39) | p=0.341 |
|  | ≥25.0 |  | 0.95 (0.79－1.15) | 0.85 (0.74－0.98) |  |
| Education level | High school | Junior high school | 0.75 (0.60－0.95) | 1.04 (0.86－1.25) | p=0.080 |
|  | College / university or higher |  | 0.76 (0.58－0.99) | 1.02 (0.84－1.24) |  |
|  | Other |  | 1.01 (0.44－2.34) | 0.35 (0.13－1.00) |  |
| Marital status | Unmarried | Married | 1.05 (0.81－1.37) | 1.01 (0.85－1.19) | p=0.668 |
| Number of household members | Alone | Two or more | 1.34 (0.94－1.90) | 0.87 (0.69－1.10) | p=0.423 |
| Work status | Unemployed | Employed | 1.20 (0.99－1.44) | 0.95 (0.83－1.10) | p=0.211 |
| Income | Less than 2,000,000 yen | More than 4,000,000 yen | 1.38 (1.08－1.77) | 1.58 (1.32－1.91) | p=0.194 |
|  | 2,000,000 to less than 4,000,000 yen |  | 1.21 (1.01－1.46) | 1.51 (1.30－1.75) |  |
| Smoking habits | Current smoker | none | 0.75 (0.62－0.91) | 1.47 (1.15－1.87) | p<0.001 |
| Drinking habits | Current drinker | none | 1.08 (0.90－1.30) | 1.01 (0.89－1.15) | p=0.357 |
| Exercise habits | No | Yes | 1.30 (1.10－1.55) | 1.36 (1.19－1.55) | p=0.909 |
| Depressive symptoms | ≥16 | <16 | 1.59 (1.30－1.96) | 1.89 (1.64－2.17) | P=0.103 |
| Insomnia | ≥6 | <6 | 1.49 (1.22－1.83) | 1.15 (0.99－1.33) | p=0.026 |
| Social capital | <9 | ≥9 | 2.31 (1.15－4.61) | 3.77 (2.28－6.23) | p=0.330 |
| Dwelling style | Prefabricated temporary housing/ Disaster restoration housing | Living in the place where they lived before the earthquake | 0.69 (0.39－1.21) | 1.04 (0.73－1.50) | p=0.833 |
|  | Rented housing |  | 1.12 (0.81－1.56) | 1.21 (0.96－1.52) |  |
|  | New homeownership after the earthquake |  | 0.89 (0.69－1.15) | 1.01 (0.82－1.24) |  |
|  | Other |  | 0.96 (0.66－1.38) | 0.91 (0.69－1.21) |  |
| House damage due to the GEJE | Damaged | Undamaged | 0.98 (0.82－1.18) | 0.89 (0.76－1.03) | p=0.489 |
| The death of family members due to the GEJE | Yes | No | 1.00 (0.64－1.57) | 1.04 (0.77－1.40) | p=0.718 |

BMI: body mass index, GEJE: great east Japan earthquake.

The interaction terms represent the effects of the variables listed in this table in combination with sex (e.g., sex*age group).

Statistical significance: P < 0.05.

Note: OR and 95% CI are shown. Extremely high or low ORs should be interpreted with caution due to potential small sample sizes or the influence of outliers. These variables were excluded from the main results table (Table 3) because they were not statistically significant (p ≥ 0.05).

**Supplemental Table 6. Multivariable-adjusted odds ratio of newly socially isolated group to not socially isolated group after multiple imputation**

|  | | Reference | Men (n = 883) | Women (n = 1,421) | Interaction term |
| --- | --- | --- | --- | --- | --- |
| Age group | 65 or more | Less than 65 | 0.62 (0.49－0.80) | 0.74 (0.65－0.86) | p=0.835 |
| Area | Coast | Inland | 0.82 (0.64－1.06) | 0.79 (0.67－1.00) | p=0.388 |
| BMI | <18.5 | ≥18.5, <25.0 | 2.09 (1.22－3.52) | 1.11 (0.87－1.40) | p=0.344 |
|  | ≥25.0 |  | 0.95 (0.79－1.17) | 0.86 (0.74－0.99) |  |
| Education | High school | Junior high school | 0.75 (0.60－0.95) | 1.04 (0.86－1.25) | p=0.080 |
|  | College / university or higher |  | 0.76 (0.58－0.99) | 1.02 (0.84－1.24) |  |
|  | Other |  | 1.01 (0.44－2.34) | 0.35 (0.13－1.00) |  |
| Marital status | Unmarried | Married | 1.05 (0.81－1.37) | 1.02 (0.85－1.20) | p=0.670 |
| Number of household members | Alone | Two or more | 1.35 (0.93－1.91) | 0.88 (0.69－1.12) | p=0.425 |
| Work status | Unemployed | Employed | 1.20 (0.99－1.44) | 0.95 (0.83－1.10) | p=0.211 |
| Income | Less than 2,000,000 yen | More than 4,000,000 yen | 1.41 (1.10－1.79) | 1.62 (1.35－1.95) | p=0.192 |
|  | 2,000,000 to less than 4,000,000 yen |  | 1.23 (1.04－1.49) | 1.54 (1.35－1.79) |  |
| Smoking habits | Current smoker | None | 0.78 (0.65－0.95) | 1.51 (1.20－1.89) | p=<0.001 |
| Drinking habits | Current drinker | None | 1.08 (0.90－1.30) | 1.01 (0.89－1.15) | p=0.357 |
| Exercise habits | No | Yes | 1.33 (1.13－1.58) | 1.39 (1.22－1.59) | p=0.918 |
| Depressive symptoms | ≥16 | <16 | 1.61 (1.33－1.99) | 1.89 (1.66－2.20) | p=0.775 |
| Insomnia | ≥6 | <6 | 1.49 (1.22－1.83) | 1.15 (0.99－1.33) | p=0.026 |
| Social capital | <9 | ≥9 | 2.34 (1.20－4.67) | 3.79 (2.30－6.25) | p=0.330 |
| Dwelling style | Prefabricated temporary housing/ Disaster restoration housing | Living in the place where they lived before the earthquake | 0.70 (0.39－1.23) | 1.08 (0.75－1.55) | p=0.836 |
|  | Rented housing |  | 1.14 (0.80－1.59) | 1.21 (0.96－1.52) |  |
|  | New homeownership after the earthquake |  | 0.89 (0.69－1.15) | 1.01 (0.82－1.24) |  |
|  | Other |  | 0.96 (0.66－1.38) | 0.91 (0.69－1.21) |  |
| House damage due to the GEJE | Damaged | Undamaged | 0.98 (0.82－1.19) | 0.90 (0.76－1.04) | p=0.490 |
| The death of family members due to the GEJE | Yes | No | 1.00 (0.64－1.57) | 1.04 (0.77－1.40) | p=0.718 |

BMI: body mass index; GEJE: Great East Japan Earthquake.
The interaction terms represent the effects of the variables listed in this table in combination with sex (e.g., sex*age group).
Results are based on the multiply imputed dataset.
Statistical significance: *P* < 0.05.
Note: ORs and 95% CIs are shown. Extremely high or low ORs should be interpreted with caution due to potential small sample sizes or the influence of outliers.

**Supplemental Table 7. The multivariable-adjusted odds ratio of the newly socially isolated group compared to the not socially isolated group by age group**

|  | |  | Men (n = 813) | | | Women (n = 1,304) | | |
| --- | --- | --- | --- | --- | --- | --- | --- | --- |
|  | | Reference | Less than 65  (n = 312) | 65 or more  (n = 501) | P for interaction | Less than 65  (n = 702) | 65 or more  (n = 602) | P for interaction |
| Area | Coast | Inland | 0.75 (0.54－1.04) | 0.76 (0.59－0.99) | p=0.980 | 0.89 (0.71－1.11) | 0.67 (0.52－0.87) | p=0.111 |
| BMI | <18.5 | ≥18.5, <25.0 | 1.96 (0.81－4.76) | 2.16 (1.12－4.18) | p=0.283 | 1.01 (0.72－1.40) | 1.26 (0.89－1.77) | p=0.111 |
|  | ≥25.0 |  | 0.82 (0.63－1.08) | 1.09 (0.89－1.33) |  | 0.97 (0.80－1.18) | 0.74 (0.60－0.91) |  |
| Education level | High school | Junior high school | 0.85 (0.51－1.40) | 1.26 (0.99－1.60) | p=0.533 | 0.84 (0.59－1.20) | 1.02 (0.82－1.27) | p=0.424 |
|  | College / university or higher |  | 0.88 (0.52－1.46) | 1.20 (0.90－1.60) |  | 0.78 (0.54－1.12) | 1.11 (0.87－1.41) |  |
|  | Other |  | 1.19 (0.29－4.83) | 0.78 (0.27－2.31) |  | 0.19 (0.03－1.43) | 0.43 (0.13－1.41) |  |
| Marital status | Unmarried | Married | 0.89 (0.62－1.27) | 1.28 (0.85－1.93) | p=0.142 | 1.07 (0.85－1.34) | 0.98 (0.76－1.27) | p=0.628 |
| Number of household members | Alone | Two or more | 0.95 (0.52－1.75) | 1.38 (0.86－2.23) | p=0.424 | 0.85 (0.59－1.23) | 0.88 (0.63－1.22) | p=0.900 |
| Work status | Unemployed | Employed | 1.32 (0.81－2.16) | 1.17 (0.95－1.44) | p=0.615 | 0.95 (0.78－1.16) | 0.94 (0.77－1.14) | p=0.748 |
| Income | Less than 2,000,000 yen | More than 4,000,000 yen | 1.93 (1.23－3.03) | 1.37 (1.00－1.87) | p=0.025 | 1.52 (1.17－1.98) | 1.65 (1.25－2.19) | p=0.569 |
|  | 2,000,000 to less than 4,000,000 yen |  | 0.98 (0.73－1.32) | 1.40 (1.09－1.81) |  | 1.39 (1.15－1.69) | 1.60 (1.25－2.05) |  |
| Smoking habits | Current smoker | None | 0.72 (0.54－0.95) | 0.76 (0.59－1.00) | p=0.718 | 1.20 (0.90－1.58) | 2.81 (1.72－4.59) | p=0.003 |
| Drinking habits | Current drinker | None | 1.05 (0.76－1.44) | 1.07 (0.86－1.34) | p=0.799 | 0.96 (0.81－1.14) | 1.11 (0.91－1.36) | p=0.270 |
| Exercise habits | No | Yes | 1.42 (1.02－1.98) | 1.25 (1.02－1.53) | p=0.474 | 1.30 (1.05－1.59) | 1.42 (1.18－1.69) | p=0.623 |
| Depressive symptoms | ≥16 | <16 | 1.94 (1.38－2.72) | 1.40 (1.08－1.83) | p=0.190 | 1.90 (1.56－2.32) | 1.88 (1.53－2.31) | p=0.948 |
| Insomnia | ≥6 | <6 | 1.30 (0.92－1.83) | 1.34 (1.26－2.11) | p=0.412 | 1.16 (0.94－1.42) | 1.13 (0.92－1.39) | p=0.740 |
| Social capital | <9 | ≥9 | 2.69 (0.85－8.57) | 2.18 (0.90－5.25) | p=0.824 | 3.08 (1.68－5.67) | 4.71 (1.92－11.53) | p=0.426 |
| Dwelling style | Prefabricated temporary housing/ Disaster restoration housing | Living in the place where they lived before the earthquake | 0.51 (0.19－1.38) | 0.83 (0.42－1.67) | p=0.485 | 1.12 (0.67－1.88) | 0.96 (0.57－1.63) | p=0.772 |
|  | Rented housing |  | 1.12 (0.75－1.68) | 1.12 (0.60－2.09) |  | 1.15 (0.88－1.51) | 1.47 (0.94－2.29) |  |
|  | New homeownership after the earthquake |  | 0.70 (0.46－1.05) | 1.06 (0.77－1.48) |  | 0.94 (0.72－1.24) | 1.11 (0.82－1.52) |  |
|  | Other |  | 1.09 (0.59－2.02) | 0.88 (0.55－1.40) |  | 0.84 (0.57－1.23) | 1.03 (0.68－1.56) |  |
| House damage due to the GEJE | Damaged | Undamaged | 0.99 (0.73－1.34) | 0.97 (0.76－1.23) | p=0.915 | 0.97 (0.70－1.12) | 0.90 (0.72－1.13) | p=0.887 |
| The death of family members due to the GEJE | Yes | No | 0.87 (0.42－1.80) | 1.08 (0.61－1.92) | p­=0.547 | 1.09 (0.74－1.60) | 1.01 (0.64－1.61) | p=0.812 |

BMI: body mass index, GEJE: great east Japan earthquake.

The interaction terms represent the effects of the variables listed in this table in combination with age group (e.g., age group*area).

Statistical significance: P < 0.05.

Note: Extremely high or low odds ratios should be interpreted with caution due to potential small sample sizes or the influence of outliers.

**Supplemental Table 8. Results for variables excluded from Table 4 due to lack of statistical significance (p ≥ 0.05)**

|  | |  | Men (n = 813) | | | Women (n = 1,304) | | |
| --- | --- | --- | --- | --- | --- | --- | --- | --- |
|  | | Reference | Undamaged  (n = 546) | Damaged  (n = 267) | P for  interaction | Undamaged  (n = 888) | Damaged  (n = 416) | P for  interaction |
| Age group | 65 or more | Less than 65 | 0.61 (0.47－0.78) | 0.56 (0.39－0.80) | p=0.759 | 0.71 (0.59－0.85) | 0.75 (0.58－0.98) | p=0.758 |
| Area | Coast | Inland | 0.75 (0.59－0.95) | 0.87 (0.55－1.38) | p=0.825 | 0.80 (0.66－0.96) | 0.87 (0.58－1.31) | p=0.697 |
| BMI | <18.5 | ≥18.5, <25.0 | 1.76 (0.93－3.34) | 3.22 (1.26－8.20) | p=0.490 | 1.15 (0.86－1.52) | 1.03 (0.66－1.58) | p=0.088 |
|  | ≥25.0 |  | 0.96 (0.79－1.17) | 0.97 (0.73－1.28) |  | 0.94 (0.79－1.11) | 0.69 (0.54－0.89) |  |
| Education level | High school | Junior high school | 1.35 (1.00－1.76) | 0.85 (0.58－1.25) | p=0.245 | 1.05 (0.84－1.31) | 1.01 (0.73－1.40) | p=0.739 |
|  | College / university or higher |  | 1.25 (0.93－1.68) | 0.98 (0.64－1.49) |  | 1.05 (0.83－1.33) | 0.97 (0.68－1.38) |  |
|  | Other |  | 1.12 (0.41－3.04) | 0.73 (0.15－3.52) |  | 0.24 (0.06－1.02) | 0.66 (0.15－2.96) |  |
| Marital status | Unmarried | Married | 1.30 (0.95－1.79) | 0.64 (0.39－1.05) | p=0.133 | 1.02 (0.83－1.25) | 0.97 (0.73－1.31) | p=0.772 |
| Number of  household members | Alone | Two or more | 1.17 (0.77－1.79) | 1.96 (1.04－3.66) | p=0.020 | 0.90 (0.67－1.20) | 0.82 (0.54－1.24) | p=0.764 |
| Work status | Unemployed | Employed | 1.19 (0.94－1.49) | 1.22 (0.88－1.69) | p=0.943 | 0.97 (0.82－1.15) | 0.92 (0.72－1.17) | p=0.678 |
| Income | Less than  2,000,000 yen | More than  4,000,000 yen | 1.23 (0.90－1.66) | 1.85 (1.22－2.81) | p=0.255 | 1.61 (1.28－2.01) | 1.54 (1.11－2.14) | p=0.141 |
|  | 2,000,000 to less than 4,000,000 yen |  | 1.16 (0.92－1.46) | 1.31 (0.94－1.82) |  | 1.66 (1.38－1.98) | 1.26 (0.97－1.63) |  |
| Smoking habits | Current smoker | none | 0.65 (0.51－0.83) | 0.96 (0.70－1.31) | p=0.042 | 1.67 (1.24－2.26) | 1.14 (1.04－1.70) | p=0.115 |
| Drinking habits | Current drinker | none | 1.11 (0.88－1.39) | 1.02 (0.75－1.38) | p=0.660 | 1.00 (0.86－1.18) | 1.05 (0.82－1.33) | p=0.887 |
| Exercise habits | No | Yes | 1.34 (1.09－1.66) | 1.23 (0.91－1.65) | p=0.489 | 1.35 (1.15－1.60) | 1.38 (1.09－1.75) | p=0.987 |
| Depressive symptoms | ≥16 | <16 | 1.49 (1.15－1.93) | 1.73 (1.23－2.44) | p=0.500 | 1.88 (1.58－2.24) | 1.90 (1.48－2.43) | p=0.914 |
| Insomnia | ≥6 | <6 | 1.47 (1.14－1.90) | 1.54 (1.09－2.17) | p=0.783 | 1.06 (0.89－1.27) | 1.33 (1.04－1.70) | p=0.221 |
| Social capital | <9 | ≥9 | 2.20 (0.89－5.44) | 2.57 (0.86－7.65) | p=0.635 | 3.60 (2.03－6.39) | 4.53 (1.55－13.22) | p=0.905 |
| Dwelling style | Prefabricated temporary housing/ Disaster restoration housing | Living in the place where they lived before the earthquake | 0.25 (0.02－2.48) | 0.70 (0.38－1.27) | p=0.440 | 0.29 (0.04－2.31) | 1.15 (0.77－1.71) | p=0.412 |
|  | Rented housing |  | 1.06 (0.71－1.57) | 1.27 (0.69－2.37) |  | 1.10 (0.84－1.44) | 1.55 (0.99－2.43) |  |
|  | New homeownership after the earthquake |  | 0.88 (0.55－1.40) | 0.93 (0.67－1.29) |  | 0.97 (0.66－1.42) | 1.05 (0.81－1.36) |  |
|  | Other |  | 0.72 (0.44－1.18) | 1.47 (0.84－2.57) |  | 0.91 (0.65－1.28) | 0.88 (0.53－1.46) |  |
| The death of family members due to the GEJE | Yes | No | 0.95 (0.44－2.09) | 1.05 (0.60－1.84) | p=0.967 | 0.83 (0.48－1.42) | 1.20 (0.66－1.58) | p=0.229 |

BMI: body mass index, GEJE: great east Japan earthquake.

The interaction terms represent the effects of the variables listed in this table in combination with house damage (e.g., house damage*age group).

Statistical significance: P < 0.05.

Note: OR and 95% CI are shown. Extremely high or low ORs should be interpreted with caution due to potential small sample sizes or the influence of outliers. These variables were excluded from the main results table (Table 4) because they were not statistically significant (p ≥ 0.05).

**Supplemental Table 9. Results for variables excluded from Table 5 due to lack of statistical significance (p ≥ 0.05)**

|  | | | Men (n = 813) | | | Women (n = 1,304) | | |
| --- | --- | --- | --- | --- | --- | --- | --- | --- |
|  | | Reference | No experience  (n = 787) | Experience  (n = 26) | P for  interaction | No experience  (n = 1,244) | Experience  (n = 60) | P for  interaction |
| Age group | 65 or more | Less than 65 | 0.60 (0.49－0.74) | 0.68 (0.16－2.86) | p=0.651 | 0.72 (0.62－0.84) | 0.81 (0.39－1.66) | p=0.953 |
| Area | Coast | Inland | 0.77 (0.63－0.95) | 0.07 (0.01－2.50) | p=0.337 | 0.80 (0.68－0.95) | 0.72 (0.07－7.56) | p=0.982 |
| BMI | <18.5 | ≥18.5, <25.0 | 2.06 (1.21－3.51) | 3.14 (0.13－7.90) | p=0.838 | 1.11 (0.87－1.41) | 1.10 (0.33－3.67) | p=0.369 |
|  | ≥25.0 |  | 0.97 (0.83－1.15) | 0.67 (0.23－1.91) |  | 0.87 (0.75－1.00) | 0.56 (0.29－1.10) |  |
| Education level | High school | Junior high school | 1.19 (0.96－1.48) | 1.16 (0.29－4.58) | p=0.441 | 1.03 (0.85－1.24) | 1.31 (0.52－3.28) | p=0.818 |
|  | College / university or higher |  | 1.19 (0.93－1.52) | 0.87 (0.17－4.55) |  | 1.02 (0.83－1.24) | 1.05 (0.39－2.83) |  |
|  | Other |  | 0.92 (0.38－2.25) | 7.91 (0.30－50.66) |  | 0.37 (0.13－1.05) | 0.37 (0.13－1.05) |  |
| Marital status | Unmarried | Married | 1.11 (0.85－1.46) | 0.34 (0.07－1.76) | p=0.265 | 1.01 (0.85－1.21) | 0.81 (0.38－1.69) | p=0.552 |
| Number of  household members | Alone | Two or more | 1.32 (0.92－1.89) | 1.61 (0.22－11.75) | p=0.915 | 0.88 (0.69－1.13) | 0.77 (0.28－2.11) | p=0.644 |
| Work status | Unemployed | Employed | 1.19 (0.98－1.44) | 1.94 (0.51－7.49) | p=0.320 | 0.98 (0.85－1.13) | 0.52 (0.26－1.03) | p=0.172 |
| Income | Less than  2,000,000 yen | More than  4,000,000 yen | 1.33 (1.03－1.70) | 5.17 (1.04－25.68) | p=0.812 | 1.57 (1.30－1.90) | 1.92 (0.75－4.92) | p=0.415 |
|  | 2,000,000 to less than 4,000,000 yen |  | 1.19 (0.99－1.44) | 2.70 (0.64－11.40) |  | 1.49 (1.28－1.73) | 2.31 (1.09－4.89) |  |
| Smoking habits | Current smoker | none | 0.75 (0.61－0.91) | 0.77 (0.25－2.41) | p=0.938 | 1.47 (1.14－1.88) | 1.25 (0.42－3.73) | p=0.824 |
| Drinking habits | Current drinker | none | 1.06 (0.88－1.27) | 2.13 (0.61－7.49) | p=0.339 | 1.02 (0.89－1.16) | 0.92 (0.47－1.82) | p=0.723 |
| Exercise habits | No | Yes | 1.26 (1.06－1.50) | 6.51 (1.66－25.52) | p=0.021 | 1.36 (1.18－1.56) | 1.60 (0.81－3.16) | p=0.492 |
| Depressive symptoms | ≥16 | <16 | 1.57 (1.28－1.94) | 2.13 (0.61－7.49) | p=0.451 | 1.90 (1.64－2.19) | 1.60 (0.80－3.20) | p=0.587 |
| Insomnia | ≥6 | <6 | 1.49 (1.21－1.83) | 1.36 (0.31－5.99) | p=0.693 | 1.11 (0.95－1.28) | 2.63 (1.32－5.24) | p=0.009 |
| Social capital | <9 | ≥9 | 2.46 (1.22－4.95) | 1.33 (0.41－4.66) | p=0.339 | 3.81 (2.27－6.40) | 2.70 (0.33－22.26) | p=0.869 |
| Dwelling style | Prefabricated temporary housing/ Disaster restoration housing | Living in the place where they lived before the earthquake | 0.67 (0.37－1.23) | 0.54 (0.07－4.18) | p=0.820 | 1.06 (0.87－1.41) | 1.22 (0.33－4.56) | p=0.531 |
|  | Rented housing |  | 1.12 (0.80－1.55) | 1.12 (0.77－1.65) |  | 1.20 (0.72－1.56) | 1.45 (0.39－5.34) |  |
|  | New homeownership after the earthquake |  | 0.91 (0.70－1.18) | 0.55 (0.15－2.06) |  | 0.95 (0.77－1.18) | 1.97 (0.84－4.63) |  |
|  | Other |  | 0.96 (0.66－1.39) | 0.30 (0.02－5.13) |  | 0.93 (0.70－1.24) | 0.35 (0.04－3.18) |  |
| House damage due to the GEJE | Damaged | Undamaged | 0.97 (0.81－1.18) | 1.25 (0.30－5.17) | p=0.645 | 0.89 (0.76－1.04) | 0.81 (0.34－1.93) | p=0.777 |

BMI: body mass index, GEJE: great east Japan earthquake.

The interaction terms represent the effects of the variables listed in this table in combination with the death of family members due to the GEJE (e.g., the death of family members due to the GEJE*age group).

Statistical significance: P < 0.05.

Note: OR and 95% CI are shown. Extremely high or low ORs should be interpreted with caution due to potential small sample sizes or the influence of outliers. These variables were excluded from the main results table (Table 5) because they were not statistically significant (p ≥ 0.05).
