# Supplementary material for: What factors are associated with new social isolation years after the great East Japan Earthquake?: findings from the TMM CommCohort study
Source: BMC Public Health. 2025 Aug 12;25:2745. doi: 10.1186/s12889-025-23778-x (PMC12341258; doi:10.1186/s12889-025-23778-x)
Supplement: Supplementary file 2 — Supplementary Material 2. [file 12889_2025_23778_MOESM2_ESM.docx]

FAMILY: Considering the people to whom you are related by birth, marriage, adoption, etc

|  | | none | one | two | three or  four | five thru  eight | nine or more |
| --- | --- | --- | --- | --- | --- | --- | --- |
| 1 | How many relatives do you see or hear from at least once a month? | ａ | ｂ | ｃ | ｄ | ｅ | f |
| 2 | How many relatives do you feel at ease with that you can talk about private matters? | ａ | ｂ | ｃ | ｄ | ｅ | f |
| 3 | How many relatives do you feel close to such that you could call on them for help? | ａ | ｂ | ｃ | ｄ | ｅ | f |

FRIENDSHIPS: Considering all of your friends including those who live in your neighborhood

|  | | none | one | two | three or  four | five thru  eight | nine or more |
| --- | --- | --- | --- | --- | --- | --- | --- |
| 1 | How many of your friends do you see or hear from at least once a month? | ａ | ｂ | ｃ | ｄ | ｅ | f |
| 2 | How many friends do you feel at ease with that you can talk about private matters? | ａ | ｂ | ｃ | ｄ | ｅ | f |
| 3 | How many friends do you feel close to such that you could call on them for help? | ａ | ｂ | ｃ | ｄ | ｅ | f |

Source: Lubben, J., Blozik, E., Gillmann, G., IIiffe, S., von Renteln Kruse, W., Beck, J. C., & Stuck, A. E. (2006). Performance of an abbreviated version of the Lubben Social Network Scale among three European Community–dwelling older adult populations. Gerontologist, 46(4), 503–513.
